# Comparative Study on the Satisfaction of Healthcare Service Providers with the Synergistic Development of Rural Healthcare Systems in China: Medical Alliance Counties vs. Non-Medical Alliance Counties

**DOI:** 10.5334/ijic.7677

**Published:** 2024-06-20

**Authors:** Meng Zhang, XiaoNan Du, GeGe Jia, QingYun Xia, YanYun Xu, Jvxiao Wu, YiLin He, Jian Wu

**Affiliations:** 1 Department of Health Management and Policy, School of Public Health, Hangzhou Normal University, Hangzhou, China; 2 Center for Project Supervision and Management National Health Commission, P.R. China, Beijing, China; 3 Department of Social Medicine and Health Management, School of Public Health, Zhengzhou University, Zhengzhou, China; 4 School of Journalism and Communication, Wuhan University, Wuhan, China

**Keywords:** vertical integration, County Medical Alliance, rural healthcare systems, synergy, professional and managerial satisfaction, China

## Abstract

**Introduction:**

This study aimed to explore whether the establishment of county medical alliances can improve satisfaction with the vertical integration of healthcare systems among rural medical and healthcare service provider managers and service providers. Our study also sought to provide recommendations for the sustainable development of vertical integration in healthcare systems.

**Methods:**

A semi-structured interview with 30 healthcare service providers was employed in this research, and Nvivo software was utilized to analyze factors that influence vertical integration. From April to July 2021, a multi-stage random sampling method was used to select participants. The sample included two leading hospitals in medical consortia, 15 member units (healthcare service providers and medical staff), two county-level hospitals, and 15 township health centers/community healthcare service centers from non-medical consortia. Questionnaire surveys were conducted with these groups. Factor analysis was used to calculate satisfaction scores for healthcare service providers with the cross-institutional synergistic development of healthcare systems in both medical and non-medical consortia (denoted as M(IQR)). Propensity score matching was employed to reduce confounding factors between groups. The Mann-Whitney U test was used to compare satisfaction differences between groups.

**Results:**

The overall satisfaction scores for lead-county hospital managers, member institution managers, medical staff at the lead-county hospital, and medical staff at member institutions were 4.80 (1.00), 4.17 (1.17), 4.00 (1.38), and 4.00 (1.12), respectively. Lead-county hospital managers’ satisfaction with cross-institutional collaboration, development capacity enhancement, and structure and resource integration in the Medical Alliance group showed higher satisfaction than the Non-Medical Alliance. Similarly, lead-county hospital medical staff in the Medical Alliance group reported greater satisfaction with collaboration efforts, supportive environment, and development capacity enhancement. Notably, while the Medical Alliance group’s satisfaction scores were higher, the differences between the two groups were not statistically significant for lead-county hospital managers and medical staff. The Medical Alliance group did show statistically significant differences in member institution managers’ satisfaction with collaboration, development capacity enhancement, and structure and resource integration. Additionally, medical staff of member institutions in the Medical Alliance group reported statistically significant higher satisfaction with collaboration, supportive environment, development capacity enhancement, healthcare service integration, and human resource development.

**Conclusion:**

To facilitate the establishment of county medical alliances, managers of leading county-level hospitals should adopt a healthcare system integration strategy. This strategy involves evolution from being a member of a single institution to a coordinator of cross-institutional vertical integration of medical and healthcare services. Additionally, revamping remuneration and appraisal systems for members of county medical alliances is necessary. This will encourage cooperation among healthcare institutions within the three-tiered system and their medical staff, ultimately facilitating the provision of integrated services.

## Introduction

In the 1990s, the emergence of managed care spurred the development of organized delivery systems, highlighting the importance of integration in the healthcare industry. This led to significant theoretical and empirical advancements [1,2]. Recognizing this, the World Health Organization (WHO) introduced a global framework for integrated healthcare services in 2016. This framework promotes horizontal and/or vertical integration of healthcare institutions to facilitate the rational allocation and effective utilization of healthcare resources. Ultimately, the goal is to ensure patients receive coordinated, preventive and curative services tailored to their specific needs across various healthcare system levels [3]. Similarly, China’s policy “Deepening Health Reform” emphasizes strengthening the responsibilities of healthcare institutions at all levels. This policy aims to achieve this by fostering technical assistance, capacity building, and the creation of a network of health institutions, ultimately promoting vertical integration of medical services [4,5].

Developed countries such as the United States, the United Kingdom, and Germany have successfully promoted the vertical integration of healthcare service institutions across all levels by reducing transaction costs based on medical service characteristics and residents’ health needs [6]. Examples include the Kaiser Group in the United States [7], the trust model in the United Kingdom [8], and disease management programs in Germany [9]. In Singapore, there are two major group models [6]. In China, vertical integration of health and medical services encompasses urban medical groups, county medical alliances, cross-regional specialty alliances, and telemedicine collaboration networks. These models aim to improve primary healthcare capacity by leveraging the strengths of prefectural and county-level hospitals and the specialized expertise of national and provincial hospitals [10].

The vertical integration of healthcare systems has strengthened the linkage and collaboration between clinical care and public healthcare services [11,12,13]. Coordination is a critical aspect of this process, as it involves collaboration among healthcare service providers, including medical institution managers and staff, at different levels [14]. Healthcare service providers are known to significantly influence the success of reform interventions [15]. Motivating healthcare professionals to participate plays a vital role in the success of vertical integration [16,17]. While the interests of medical and healthcare service providers (including managers and professionals) at different levels may vary, they also share common purpose. This allows for smooth communication, information sharing [18], improved patient satisfaction, and increased access to medical services [19,20]. In other words, vertical integration requires the development and maintenance of a common frame of reference (shared mission, vision, values, and culture) across organizations, professional groups, and individuals, a concept known as normative integration [21].

The impact of vertical integration on healthcare service systems remains a topic of debate among scholars. Some argue that it leads to higher prices, increased costs, and no improvement in quality [22,23,24,25,26]. Others suggest it could reduce healthcare spending and enhance the quality of care by facilitating communication across care settings and reducing unnecessary and low-value care [23,27,28]. Previous research has examined the impact of vertical integration on primary healthcare professionals within primary healthcare institutions, including physician compensation, autonomy versus system support, medical professionalism and culture, and other relevant factors [22]. A study by Shasha Yuan, Fengmei Fan, and Joris van de Klundert explored the perspective of primary healthcare professionals on the vertical integration of the healthcare system in China [28]. However, the interests and satisfaction of medical and healthcare service providers involved in the cross-institutional synergistic development of healthcare systems remain largely underexplored.

Currently, the most common tool for evaluating integrated healthcare services is the “structure-process-outcome” model proposed by Devers in 1994. This model assesses healthcare service integration across three dimensions: structural measurement (medical and healthcare service integration), process measurement (the intermediate steps taken to achieve the desired outcome), and outcome measurement (the degree to which the ultimate goal is achieved) [29]. Building on this framework, this study dissects the interests of medical and healthcare service providers involved in the vertical integration of healthcare services into three key aspects: integration structure (focusing on the cross-institutional synergistic structure), integration process (examining resource integration), and integration outcome (investigating the collaborative development capacity of medical institutions).

A joint study titled “Report Recommends Deeper Healthcare Reforms in China” was conducted by the World Bank, the World Health Organization, the Ministry of Finance, the National Health and Family Planning Commission, and the Ministry of Human Resources and Social Security. This report recommended transforming China’s healthcare system into a people-oriented and quality-oriented integrated service delivery system with strong grassroots healthcare services as the foundation. This model, called “people-oriented integrated care,” includes eight core action areas [4]. Vertical integration, one of these core areas, focuses on three key aspects: defining facility roles within the integrated network, establishing provider-to-provider relationships, and forming facility networks [4,5]. Establishing county medical alliances is a crucial step in addressing the fragmentation of rural healthcare and a viable solution to promote vertical integration of rural healthcare services in China [30,31,32]. These alliances are policy-oriented health organizations led by local governments [33]. Additionally, they play a vital role in alleviating the phenomenon of “tertiary hospitals overcrowded and grassroots hospitals deserted” [34].

The county medical alliance, led by county-level hospitals and supported by lower-tier healthcare institutions, aims to optimize the allocation of county healthcare resources, restructure service processes, and enhance the healthcare system to create a three-tiered linked and synergistically developed model [35]. This model includes two structures: a less structured medical alliance and a compact medical alliance. The compact medical alliance operates under a direct management model. The leading hospital director serves as the sole legal representative, utilizing technology, management expertise, and assets to achieve a partial integration of property rights. This model grants the leading hospital responsibility for administrative management and business operations across personnel, finance, and health resources of member institutions [36,37]. While member units maintain their organizational setup and administrative framework, and continue to fulfill basic medical care and public health service functions with existing financial mechanisms, the leading hospital implements integrated management across various domains. These domains include human resources, medical services, financial systems, performance evaluations, resource allocation, centralized procurement, information technology development, and prepayment of medical insurance. This multifaceted approach aims to enhance service capabilities at the grassroots level and improve the current state of basic medical and healthcare services in rural areas [38]. However, no scholars have hitherto investigated the impact of constructing a compact medical alliance on the satisfaction of healthcare service providers regarding cross-institutional synergistic development.

By understanding healthcare service providers’ satisfaction with the cross-institutional collaboration fostered by vertical integration, we can gain valuable insights into their role in shaping implementation efforts and the overall impact of this integration [15]. This study, therefore, investigated the impact of county medical alliances on the satisfaction of healthcare service providers during the process of medical and healthcare service integration. By identifying an equilibrium point for their satisfaction, the study sought to provide insights to strengthen the continuity of labor division and cooperation between different levels of rural medical institutions and ultimately promote coordination, improve overall health outcomes, and enable the sustainable development of the entire rural medical service system. Additionally, this study sought to provide recommendations for the sustainable development of vertical integration in healthcare systems, particularly in developing countries.

## Methods

### Study Design

A multi-stage random sampling method was employed to select both the study sites and research subjects. In the first stage, Anhui and Fujian provinces were chosen as the study locations. These provinces were designated as project provinces under The World Bank China Health Reform Program-for-Results (the XII health project), which aligns with the goals of this study by complementing and promoting the implementation of China’s health reform plan. In the second stage, factors such as economic level, county-rural integration status, geographical distribution, and regional support were all carefully considered. Subsequently, Suixi County in Anhui and Youxi County in Fujian were randomly selected from pilot counties with over three years of medical alliance implementation. These counties became the intervention implementation samples, chosen using the random sample table method. Shanghang County in Fujian, a county without a medical alliance, was chosen as the control group. Notably, all three sample counties have similar healthcare population characteristics and healthcare service levels. Next, the study sites encompassed the lead county-level health institutions within the sample county medical alliance. Additionally, 15 township health institutions/community health centers and their corresponding village clinics/community health clinics were randomly selected from the sample medical alliance counties. The same selection process (15 institutions and corresponding clinics) was applied in the sample non-medical alliance county, selecting 2 county-level health institutions and their corresponding township health centers/community health centers. Finally, the list of managers and medical staff of medical institutions in the study site was collected in advance, and 90 managers of county-level medical institutions, 450 managers of primary medical and health service institutions, 330 county-level medical personnel and 1,200 medical personnel of primary medical institutions were randomly selected by systematic sampling method. Inclusion criteria for respondents: (1) participate in or be familiar with the vertical integration of rural medical and health service system; (2) managers and medical staff who have worked in the unit for more than 3 years; exclusion criteria: (1) those who are not on duty during the investigation; and (2) refuse partners.

### Healthcare service providers consultations

For this study, we first defined and categorized healthcare service providers involved in the synergistic development of vertical integration within county healthcare systems. These categories included county-level health institutions/lead county hospitals and their medical staff and primary healthcare institutions/member institutions and their medical staff. To develop the interview outline, we analyzed relevant documents, including policies and strategies for synergistic development in healthcare systems, the synergistic development model itself, elements and effects of vertical integration in county healthcare systems, and the interests of healthcare service providers on the supply side of healthcare services (Supplementary Table 1 and Supplementary Table 2 for details). We conducted semi-structured interviews with a total of 30 healthcare service providers. In the sample counties with medical alliances, we interviewed two people in charge from each lead county hospital and member institution, three people in charge of relevant departments from each, and five medical staff members from each. In the non-medical alliance county, we followed the same structure, interviewing two people in charge from each county-level and primary healthcare institution, three people in charge of relevant departments, and five medical staff members.

### Questionnaire Design

To assess healthcare service providers’ satisfaction with the synergistic development of vertical integration, we developed separate sets of structured questionnaires tailored to different groups. Managers from county-level health institutions/lead county hospitals received an 8-question survey, while managers from primary healthcare institutions/member institutions received a 14-question survey. Medical staff from both groups participated in a 24-question survey. The questionnaires addressed key aspects of vertical integration across three dimensions: structure, process, and outcomes. The integration structure dimension focused on governance institutions, healthcare service institution resources, and the integration of resource allocation and technological configuration. The integration process section explored the establishment of a healthcare service referral system, mutual exchange and recognition of business information, and the seamless continuity of bidirectional referral prescriptions. Finally, the integration outcomes covered the influential role of the leading hospital and the improvement of diagnostic and treatment capabilities within healthcare institutions. Each question utilized a 5-point Likert scale, ranging from 1 (not at all satisfied) to 5 (very satisfied). Trained surveyors conducted on-site surveys in the three sample counties between April and July 2021. All completed questionnaires were carefully reviewed, and only those meeting the criteria were considered valid.

### Study Population

Eighty-nine managers from Lead County hospitals participated in the survey (31 in the control group and 58 in the treatment group). This sample exhibited a mild male predominance (51%) with an average age of 41.65 years. Most respondents (79%) were permanent employees, averaging 18.78 years of service (Supplementary Table 7 for details).

448 managers from member institutions participated in the survey as respondents, including 89 in the control group and 359 in the treatment group. Of the respondents, 63% were male, with an average age of 43.75 years. They reported being in good health (96%), and 68% were permanent employees. Their average length of service was 18.71 years (See Supplementary Table 8 for details).

A total of 302 medical staff members from hospitals in Lead County participated in the survey. These participants were divided into two groups: a control group (n = 80) and a treatment group (n = 222). The survey subjects were nearly half male (49%), with an average age of 35.81 years. Self-reported health status indicated that 98% of the medical staff were moderately healthy. Additionally, 60% were permanent employees, averaging 12.05 years of service. (Detailed basic demographic characteristics are provided in Supplementary Table 9).

A total of 1,093 medical staff working in member institutions were interviewed, with 285 in the control group and 808 in the treatment group. The survey subjects exhibited a female predominance (56%) with a mean age of 38.83 years. Additionally, 96% reported moderate health, 79% were permanent employees, and the average length of service was 14.58 years. (Detailed basic demographic characteristics are detailed in Supplementary Table 10).

### Data analysis

Qualitative data were obtained by conducting and recording all interviews, which were then transcribed into Word documents and analyzed using NVivo 12 Pro software. The data were systematically organized and coded according to a three-stage process: open coding, axial coding, and selective coding. Theoretical results were continually compared and revised until data saturation was achieved. This three-level coding process not only allows analysis of the relationship between core and main category types but also reveals the relationship between various elements. The results were finally summarized in an Excel worksheet.

The quantitative data were uploaded into EpiData software to create a database, where data entry and data cleaning were completed by double entry. Statistical analyses were conducted using SPSS 26.0 and STATA 16.0 software. The Cronbach’s alpha coefficient was used to test the reliability of the questionnaire, and factor analysis was used to test the structural validity. Satisfaction scores for each dimension and the overall satisfaction score were calculated based on the factor score coefficient matrix and the variance explained by factors [39,40], denoted as M(IQR). 1:2 propensity score matching (a technique to minimize group differences) was used to minimize the difference in confounding factors between the medical alliance group and the non-medical alliance group. The Mann-Whitney U test was used to compare differences in satisfaction with medical and healthcare service providers between the medical alliance group and the non-medical alliance group. A two-sided p-value < 0.05 was considered statistically significant.

### Variables

In the propensity score matching process, we treated the presence of a medical alliance in the sample counties as the independent variable. Counties with a medical alliance were coded as 1, and those without were coded as 0. The dependent variables were the overall satisfaction score and the satisfaction score for each factor (all ranging from 1 to 5). Additionally, basic demographic characteristics such as gender, age, and marital status were included as covariates to account for potential confounding factors.

### Ethical approval

The study protocol was reviewed and approved by Hangzhou Normal University’ scientific research ethics committee. Before each interview, the first author introduced the study’s objectives and obtained verbal consent and permission to record the interview from the participants.

## Results

### Interview results

**Figure 1:**
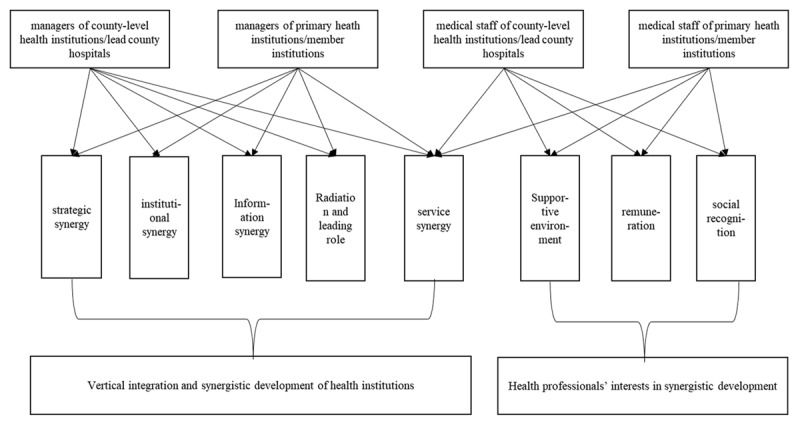
Interests of healthcare service providers in Cross-institutional Synergistic Development.

In this study, we classified and integrated categories based on their conceptual connections and logical sequences. We employed a three-level coding system to extract a more comprehensive core category from the resultant main categories. The open coding process yielded 23 initial concepts. During the following main axis coding, we analyzed the 23 initial concepts by comparing them and considering the hierarchical relationship among them to find the main generic category (main category) and secondary generic categories (subcategories). This analysis revealed that 33 construction strategies can be summarized into 8 subcategories and 3 main categories (See Supplementary Table 11). The qualitative analysis also highlighted the shared values, norms, and goals of healthcare service providers during vertical integration in healthcare service systems. At the individual level, medical staff hoped to obtain a supportive environment, good remuneration, and social recognition to realize service synergy among intra-county health institutions and improve the continuity of intra-county healthcare services. Managers of health institutions expected that institutional synergy, strategic synergy, and information synergy among health institutions across all levels of the healthcare system could achieve profession-level integration. This included playing the role of an external radiation source as the lead institution, strengthening the critical care treatment capacity of the lead institution, and enhancing the service capacity of primary healthcare institutions. Finally, at the organization level, the goal was to achieve service synergy among intra-county health institutions and increase intra-county visiting rates (Figure 1).

### Analysis of satisfaction of healthcare service providers with synergistic development

#### Managers of lead county hospitals

The Cronbach’s alpha coefficient of the questionnaire was 0.948, indicating high reliability. Additionally, the Kaiser-Meyer-Olkin (KMO) value of 0.918 and Bartlett’s test of sphericity chi-square value of 598.363 (p < 0.001) confirmed the suitability of the data for factor analysis. Principal component analysis with maximum variance and orthogonal rotation was used to extract two factors from the eight structured questions: factor 1, satisfaction with cross-institutional synergistic development capacity enhancement, and factor 2, satisfaction with cross-institutional synergistic structure and resources integration. (The details of the factor analysis and satisfaction score calculation process are provided in Supplementary Table12).

Propensity score matching resulted in 48 managers in the lead unit of the medical alliance group and 27 managers in the non-medical alliance group. Detailed results on the sample matching effectiveness are provided in Supplementary Table 13. The overall satisfaction score with cross-institutional synergistic development, satisfaction with cross-institutional synergistic development capacity enhancement, and satisfaction with cross-institutional synergistic structure and resource integration were 4.80(1.00), 4.80(1.00), and 4.36(1.00), respectively. Table 1 showed that the satisfaction scores of the medical alliance group were higher than those of the non-medical alliance group, but there was no statistically significant difference between the two groups.

**Table 1:** Satisfaction scores of Lead County Hospital Managers.


GROUP	SAMPLE SIZE	OVERALL SATISFACTION WITH CROSS-INSTITUTIONAL SYNERGISTIC DEVELOPMENT	SATISFACTION WITH CROSS-INSTITUTIONAL SYNERGISTIC DEVELOPMENT CAPACITY ENHANCEMENT	SATISFACTION WITH CROSS-INSTITUTIONAL SYNERGISTIC STRUCTURE AND RESOURCE INTEGRATION

medical alliance group	48	4.85(0.77)	4.82(0.79)	4.65(1.00)

non-medical alliance group	27	4.38(1.00)	4.40(1.00)	4.35(1.00)

Z value		–1.224	–0.902	–1.321

P-value		0.221	0.367	0.186


### Managers of member institutions

The Cronbach’s alpha coefficient of the questionnaire was high (0.979), indicating strong reliability. The KMO value of 0.963 and Bartlett’s test of sphericity chi-square value of 5938.346 (p < 0.001) confirmed the data’s suitability for factor analysis. Two factors were extracted from the 14 questions: factor 1, satisfaction with cross-institutional synergistic development capacity enhancement, and factor 2, satisfaction with cross-institutional synergistic structure and resource integration. (Detailed results on factor analysis are provided in Supplementary Table 14).

Propensity score matching resulted in 281 managers in member institutions of the medical alliance group and 85 managers in the non-medical alliance group. Detailed results on the sample matching effectiveness are provided in Supplementary Table 15. The overall satisfaction score with cross-institutional synergistic development, satisfaction with cross-institutional synergistic development capacity enhancement, and satisfaction with cross-institutional synergistic structure and resource integration were 4.17(1.17), 4.20(1.11), and 4.00(1.28), respectively. Table 2 showed that the satisfaction scores of the medical alliance group were higher than those of the non-medical alliance group, and this difference was statistically significant.

**Table 2:** Satisfaction Scores of Member Institution Managers.


GROUP	SAMPLE SIZE	OVERALL SATISFACTION WITH CROSS-INSTITUTIONAL SYNERGISTIC DEVELOPMENT	SATISFACTION WITH CROSS-INSTITUTIONAL SYNERGISTIC DEVELOPMENT CAPACITY ENHANCEMENT	SATISFACTION WITH CROSS-INSTITUTIONAL SYNERGISTIC STRUCTURE AND RESOURCE INTEGRATION

medical alliance group	281	4.33(1.10)	4.36(1.00)	4.02(1.23)

non-medical alliance group	85	4.01(1.72)	4.00(1.67)	4.00(2.00)

Z value		–2.590	–2.839	–2.023

P-value		0.010***	0.005***	0.043**


*Note*: ** P < 0.05, *** P < 0.01.

### The medical staff of lead county hospitals

The Cronbach’s alpha coefficient of the questionnaire was high (0.987), indicating strong reliability. The KMO value of 0.971 and Bartlett’s test of sphericity chi-square value of 11137.383 (p < 0.001) confirmed the data’s suitability for factor analysis. Two factors were extracted from the 24 questions: factor 1, satisfaction with supportive environment and human resources development, and factor 2, satisfaction with cross-institutional synergistic development capability enhancement (Detailed results on factor analysis are provided in Supplementary Table 16).

Propensity score matching resulted in 217 medical staff in lead hospitals of the medical alliance group and 77 medical staff in the non-medical alliance group. Detailed results on the sample matching effectiveness are provided in Supplementary Table 17. The overall satisfaction score with cross-institutional synergistic development, satisfaction with a supportive environment and human resources development, and satisfaction with cross-institutional synergistic development capability enhancement were 4.00(1.38), 4.00(1.52), and 4.14(1.26), respectively. Table 3 showed that the satisfaction scores of the medical alliance group were higher than those of the non-medical alliance group, but there was no statistically significant difference between the two groups.

**Table 3:** Satisfaction Scores of Medical Staff in the Lead County Hospitals.


GROUP	SAMPLE SIZE	OVERALL SATISFACTION WITH CROSS-INSTITUTIONAL SYNERGISTIC DEVELOPMENT	SATISFACTION WITH A SUPPORTIVE ENVIRONMENT AND HUMAN RESOURCES DEVELOPMENT	SATISFACTION WITH CROSS-INSTITUTIONAL SYNERGISTIC DEVELOPMENT CAPABILITY ENHANCEMENT

medical alliance group	217	4.01(1.42)	4.00(1.57)	4.22(1.32)

non-medical alliance group	77	3.90(1.27)	4.00(1.41)	4.00(1.19)

Z value		–0.35	–0.46	–0.01

P-value		0.729	0.646	0.992


### Medical Staff of Member Institutions

The Cronbach’s alpha coefficient (0.985) indicated high questionnaire reliability. The KMO value of 0.981 and Bartlett’s test of sphericity chi-square value of 35826.057 (p < 0.001) confirmed the data’s suitability for factor analysis. Four factors were extracted from the 24 questions: factor 1, identified as satisfaction with a supportive environment; factor 2, satisfaction with cross-institutional synergy capacity enhancement; factor 3, satisfaction with healthcare service integration; and factor 4, satisfaction with human resource development. (Detailed results on factor analysis are provided in Supplementary Table 18).

Propensity score matching resulted in 764 managers in member institutions of the medical alliance group and 276 managers in the non-medical alliance group. Detailed results on the sample matching effectiveness are provided in Supplementary Table 19. Scores included: satisfaction with cross-institutional synergy (4.00(1.12)), satisfaction with a supportive environment (4.00(1.26)), satisfaction with cross-institutional synergistic development capacity enhancement (4.00(1.26)), satisfaction with healthcare service integration (4.00(1.37)), and satisfaction with human resource development (4.00(1.67)).The overall satisfaction scores for the medical alliance group were higher than those of the non-medical alliance group, and this difference was statistically significant (Table 4).

**Table 4:** Satisfaction scores of the Medical Staff in Member Institutions.


GROUP	SAMPLE SIZE	OVERALL SATISFACTION WITH CROSS-INSTITUTIONAL SYNERGISTIC DEVELOPMENT	SATISFACTION WITH A SUPPORTIVE ENVIRONMENT	SATISFACTION WITH CROSS-INSTITUTIONAL SYNERGISTIC DEVELOPMENT CAPACITY ENHANCEMENT	SATISFACTION WITH HEALTHCARE SERVICE INTEGRATION	SATISFACTION WITH HUMAN RESOURCE DEVELOPMENT

medical alliance group	764	4.00(1.28)	4.00(1.32)	4.00(1.38)	4.00(1.37)	4.00(1.70)

non-medical alliance group	276	3.93(0.88)	3.92(0.77)	3.87(1.00)	3.86(1.14)	3.82(1.00)

Z value		–4.90	–4.81	–5.14	–4.84	–4.13

P-value		P < 0.001***	P < 0.001***	P < 0.001***	P < 0.001***	P < 0.001***


*Note*: *** P < 0.01.

## Discussion

The establishment of medical alliances, widely advocated by the National Health Commission of China as a primary method for achieving people-centered integrated care, is an effective way to optimize the allocation of medical and health resources [41,42]. Healthcare service providers play a critical role in the successful vertical integration of the healthcare service system. Analyzing their satisfaction regarding vertical integration can provide insights into their role [16]. Therefore, this study examined the impact of constructing Medical Alliances on healthcare service providers’ satisfaction with the vertical integration of healthcare systems. The aim was to identify an optimal level of satisfaction that promotes enhanced collaboration among healthcare institutions at different levels, further enhancing the continuity, coordination, and precision of rural healthcare services.

The results showed that establishing a medical alliance did not significantly improve the satisfaction level of managers and medical staff in lead county hospitals regarding inter-institutional collaboration. During medical alliance integration, lead county hospitals need to invest significant manpower, material resources, and financial resources. This shift towards vertical integration imposes a significant demand on managers and staff accustomed to working in a standalone institution. They must learn about and understand the complexities and culture of running a multi-agency system [43]. This additional burden may be one reason why healthcare providers in lead county hospitals did not report increased satisfaction with inter-institutional collaboration. Furthermore, studies conducted in the United States and China from the perspectives of physicians and primary healthcare professionals point out that higher-level hospitals face growing pressure during vertical integration, particularly regarding shared goals, vision, and leadership [22,28]. Interviews with some managers from lead county hospitals support these findings. They mentioned a significant workload increase after the medical alliance’s development process. One county-level hospital manager stated, “*With the establishment of the county medical alliance, our responsibilities involve not only our hospital’s development but also implementing systems and documents related to the medical alliance’s development. Additionally, we must provide technical guidance to primary healthcare institutions. This has certainly increased our workload. The medical alliance’s development process has not only increased our responsibilities but also demanded higher capabilities in comprehensive management and service”*.

Previous studies on integrated care reform in urban China also yielded similar findings. Some medical staff in tertiary hospitals reported feeling overburdened due to increased workloads after participating in integrated care, with no clear policies to compensate them for additional service provision [43,44,45]. This concern aligns with the findings of this study. County-level healthcare institutions, while assisting in the development of grassroots facilities, may experience a weakening of their own service capabilities [46]. This lack of improvement in service capacity could potentially contribute to the unchanged satisfaction regarding inter-institutional collaboration among lead healthcare providers.

Furthermore, interviews with medical staff in county-level hospitals supported these concerns. One staff member mentioned, “*The workload significantly increased after joining the medical alliance. Our duties include not only our daily medical tasks but also regular visits to lower-level facilities to provide training to primary healthcare practitioners. The delay in reforming the remuneration system to compensate for this increased workload has contributed to dissatisfaction*”.

Several studies have suggested that collaborating with higher-level hospitals can benefit county-level institutions during rural healthcare integration. One study found that county-level hospitals improved management, service capacity, and business volume, even developing new medical technologies, with the help of urban tertiary hospitals [46]. Another research highlights financial incentives as a key motivator for changing physician behavior [47]. Therefore, it is recommended that county-level healthcare institutions establish medical alliances with city or provincial-level hospitals during vertical integration. This collaboration can enhance their own healthcare service capabilities, ultimately elevating the overall quality and capacity of the rural healthcare system. Furthermore, a scientifically designed performance evaluation system is crucial. This system should incorporate key indicators such as the number of regular visits to primary care facilities, clinical teaching rounds conducted, and onsite technical assistance provided. By increasing the proportion of performance-based pay in the reward system, medical staff in leading hospitals will be motivated to actively participate in supporting the development of primary care.

The satisfaction analysis showed that constructing medical alliances significantly improved managers’ and medical staff’s satisfaction within member institutions regarding both cross-institutional synergistic development and capacity enhancement for such development. This finding aligns with research on vertical integration between hospitals and physicians, which demonstrates improved coordination and communication frequency among healthcare institutions at all levels [26,27,48]. These studies also highlight the benefits of implementing various methods, such as technical assistance, which enhance information sharing and improve the quality and efficiency of services provided by primary healthcare institutions. Similar to a previous study where medical staff reported satisfaction with ward rounds and clinical teaching organized by leading hospitals [37], this study found that constructing medical alliances strengthens the connection and communication between leading hospitals and member institutions. This increased opportunity for participation in relevant activities likely contributed to improved satisfaction among healthcare providers in grassroots institutions.

A manager from a township hospital echoed these sentiments, stating, “*The formation of a medical alliance has significantly enhanced the service capacity and organizational performance of member institutions. This improvement is largely due to the radiating effect of lead county hospitals. Additionally, the integration of healthcare resources across different levels, regular visits by senior physicians to rural areas, and seamless information exchange within the medical alliance have all contributed to this positive development*”.

This study’s findings align with previous research highlighting the positive impact of vertical integration on primary healthcare professionals [28,45]. By enhancing the work environment and career advancement opportunities at primary healthcare institutions, medical staff experience greater job satisfaction and fulfillment. Additionally, their expertise, service capabilities, and overall service quality improve, leading to a stronger sense of achievement. Another study found that vertical integration increases training opportunities for primary care providers, allowing them to upskill and potentially move up within the larger organization [12]. This aligns with the present research, where constructing medical alliances improves satisfaction with environmental support and human resource development among healthcare providers in grassroots institutions.

A medical staff member from a township hospital corroborated these improvements, stating, “*The infrastructure and living facilities of primary healthcare institutions have been significantly improved, which better meets the needs of medical staff for a comfortable work environment. Additionally, medical personnel now have more opportunities for continuing education, such as training and advanced studies. This fulfills their needs for professional development, allowing them to improve their professional and technical abilities and ultimately increasing their sense of accomplishment*”.

Furthermore, research on vertical integration extends beyond its impact on staff satisfaction. A study examining access to surgical care for Medicaid beneficiaries found that vertical integration is associated with increased Medicaid acceptance rates among practices, allowing greater access to surgical care for vulnerable, low-income patients [49]. Similarly, other studies suggest that vertical integration has the potential to reduce hospital readmissions [50,51]. These findings indirectly confirm that vertical integration of healthcare services significantly enhances the capabilities of grassroots institutions, ultimately improving accessibility to medical care for residents at the primary level.

Effective communication is crucial for successful vertical integration in healthcare. Clear communication of the integration strategy, along with fostering involvement and collaboration at all levels of the institution, increases the chances of a smooth transition [52]. Additionally, improving job satisfaction among primary healthcare personnel is vital. This can be achieved through better pay and benefits, but also by providing more opportunities for career development [53]. Therefore, several recommendations can be made to enhance vertical integration. First, utilizing information technology can further strengthen communication and collaboration among healthcare institutions at all levels. This could include promoting mutual recognition of examination results within the medical alliance, streamlining the process for residents seeking medical care across institutions, and reinforcing the continuity of healthcare services. Second, during integration, it is important to motivate healthcare personnel in primary healthcare institutions. While competitive compensation is important, non-economic incentives such as opportunities for professional title promotion and training can be equally effective.

## Conclusions

This study identified significant achievements in synergistic development among healthcare institutions within the sampled medical alliance county. Notably, member institutions’ capacity for synergistic development improved considerably. However, managers and medical staff at lead county hospitals did not report a significant increase in satisfaction with cross-institutional collaboration. This could be attributed to the additional complexity and workload associated with their role. To address this, health administrative departments should guide lead county hospital managers to adopt a holistic approach to healthcare system development and support their role transformation during vertical integration. Additionally, reforming and improving remuneration and appraisal mechanisms is crucial. This can incentivize collaboration between stakeholders, boost medical staff motivation, and ultimately enhance satisfaction with cross-institutional synergistic development. Consequently, this will promote successful vertical integration of healthcare institutions at all levels within a county. Furthermore, it is recommended that lead county hospital managers embrace a healthcare system integration development concept. This shift requires transitioning from solely organizing institutional services to actively orchestrating cross-institutional vertical integration of healthcare services.

## Additional File

The additional file for this article can be found as follows:

10.5334/ijic.7677.s1 Supplementary Tables. Tables 1 to 19.
