# Supplementary material for: Comparative Study on the Satisfaction of Healthcare Service Providers with the Synergistic Development of Rural Healthcare Systems in China: Medical Alliance Counties vs. Non-Medical Alliance Counties
Source: Int J Integr Care. 2024 Jun 20;24(2):26. doi: 10.5334/ijic.7677 (PMC11192093; doi:10.5334/ijic.7677)
Supplement: Supplementary Tables. — Tables 1 to 19. [file ijic-24-2-7677-s1.pdf]

Supplementary Table 1 Outline for Interviews on the Synergistic Development of County-level Healthcare Service Systems of Management Personnel of Medical Institution

No.	Interview Questions
**1**	What achievements have been made in the process of vertical integration in the healthcare service system in terms of systematic coordinated development? What are the shortcomings?
**2**	What specific systemic coordinated development interests and demands do you have regarding the vertical integration of the healthcare service system? Have they been met?
**3**	In the process of promoting vertical integration in the healthcare service system, have relevant regulations or agreements been formulated to clarify the responsibilities, rights, and interests relationships of healthcare service institutions at various levels? If so, please provide specific details.
**4**	In the process of vertical integration in the healthcare service system, have mechanisms for sharing benefits been established among healthcare service institutions at various levels? If so, please provide specific details.
**5**	In the process of vertical integration in the healthcare service system, what measures have been taken to facilitate the upward and downward mobility of healthcare service personnel?
**6**	In the process of vertical integration in the healthcare service system, have assessment and incentive mechanisms been established to promote the integration and decentralization of medical resources? If so, please provide specific details.
**7**	In the process of vertical integration in the healthcare service system, have planning systems and mechanisms been formulated to adapt to the talent needs at the grassroots level and the different development of talents in various regional medical institutions? If so, please provide specific details.
**8**	Have reasonable mechanisms for the flow of talent and professional collaboration been established for cooperation and collaboration among medical institutions within the healthcare service system? If so, please provide specific details.
**9**	Have guidelines or systems been formulated to guide the healthcare service system in providing continuous services for patients, including diagnosis, rehabilitation, and long-term care? If so, please provide specific details.
**10**	Have communication and exchange systems been established for the referral of patients among vertically integrated medical institutions? If so, please provide specific details.
**11**	In the process of vertical integration in the healthcare service system, have standardized management systems for healthcare information systems been established? If so, please provide specific details.
**12**	In your opinion, what aspects of systemic coordinated development should be the main focus in the process of vertical integration in the healthcare service system?

Supplementary Table 2 Outline for Interviews on the Synergistic Development of County-level Healthcare Service Systems of Medical Service Personnel in Healthcare Institutions

No.	Interview Questions
**1**	What achievements have been made in the process of vertical integration in the healthcare service system in terms of systematic coordinated development? What are the shortcomings?
**2**	What specific systemic coordinated development interests and demands do you have regarding the vertical integration of the healthcare service system? Have they been met?
**3**	In the process of vertical integration in the healthcare service system, what methods have been employed to improve trust and commitment among healthcare institutions as service providers? If so, please provide specific details.
**4**	Have good diagnostic and collaborative management orders and systems been established among other healthcare institutions? If so, please provide specific details.
**5**	Has a scientifically reasonable promotion system been provided in the process of vertical integration? If so, please provide specific details.
**6**	Have mechanisms for promoting the sharing of interests in the vertical integration been established? If so, please provide specific details.
**7**	In the process of vertical integration, is there a positive collaborative organizational culture? If so, please provide specific details.
**8**	Is there a mechanism for the smooth exchange of information established? If so, please provide specific details.
**9**	Do all levels of healthcare service institutions have clearly defined responsibilities in the process of providing healthcare services?
**10**	Have mechanisms for efficient information connectivity been established? If so, please provide specific details.
**11**	In the process of vertical integration, is there a culture of sharing knowledge (technical, informational, etc.)? If so, please provide specific details.
**12**	In your opinion, what aspects of systemic coordinated development should be the main focus in the process of vertical integration in the healthcare service system?

Supplementary Table 3 The Questionnaire of Lead County Hospital Managers

Survey item	Likert item				
X1 Establish a referral system for healthcare services	1	2	3	4	5
X2 Mutual transmission, circulation and recognition of business information	1	2	3	4	5
X3 Guarantee continuity of two-way referral medications	1	2	3	4	5
X4 Bring into play the radiation role of lead hospitals//county-level health institutions	1	2	3	4	5
X5 Improve emergency and critical care capability of lead hospitals/county-level health institutions	1	2	3	4	5
X6 Establish unified governance organization or coordination department	1	2	3	4	5
X7 Adjust and train human resources in timely manner along with health institution development	1	2	3	4	5
X8 Adjust and allocate health facilities and technologies among health institutions in unified manner based on resources allocation plan and business development plan	1	2	3	4	5

Supplementary Table 4 The Questionnaire of Managers in Primary Health Institutions

Survey item	Likert item				
X1 Bring into play the radiation role of lead hospitals/county-level health institutions	1	2	3	4	5
X2 Improve emergency and critical care capability of health institutions at this level	1	2	3	4	5
X3 Awareness of holism and collaboration	1	2	3	4	5
X4 Integrated-service concept and group consciousness	1	2	3	4	5
X5 Establish and improve Benefit-sharing mechanism	1	2	3	4	5
X6 Guarantee continuity of two-way referral medications	1	2	3	4	5

X7 Improve organizational performance of member institutions/primary health institutions	1	2	3	4	5
X8 Mutual transmission, circulation and recognition of business information	1	2	3	4	5
X9 Establish healthcare referral system	1	2	3	4	5
X10 Adjust capital investment in timely manner in line with development needs of health institutions	1	2	3	4	5
X11 Adjust government financial inputs in timely manner in line with the development needs of health institutions at all levels	1	2	3	4	5
X12 Adjust and allocate health facilities and technologies among health institutions in unified manner based on resources allocation plan and business development plan	1	2	3	4	5
X13 Adjust and train human resources in timely manner along with health institution development	1	2	3	4	5
X14 Establish unified governance organization or coordination department, improve organizational structure and collaboration mechanism, and form unified governance structure	1	2	3	4	5

Supplementary Table 5 The Questionnaire of Medical Staff in County Hospitals

Survey item	Likert item				
X1 Provide necessary living facilities for medical staff	1	2	3	4	5
X2 Good personal development obtained by medical staff	1	2	3	4	5
X3 More training provided to medical staff to improve their abilities	1	2	3	4	5
X4 Preferable security, medical dispute compensation and roadside insurance provided for medical staff	1	2	3	4	5
X5 Opinions of medical staff can be valued when decisions involving the interests of medical staff	1	2	3	4	5
X6 Increase the remuneration level for medical staff	1	2	3	4	5

X7 Medical staff from multiple institutions all can be supported and respected when providing health services synergistically	1	2	3	4	5
X8 Provide necessary working conditions for medical staff	1	2	3	4	5
X9 Opinions of medical staff can be valued when making decisions on important matters	1	2	3	4	5
X10 Good organizational culture and atmosphere	1	2	3	4	5
X11 Good doctor-patient relationship	1	2	3	4	5
X12 Scientific and reasonable promotion system provided for medical staff	1	2	3	4	5
X13 Reduce medical burden of patients at grass-roots level	1	2	3	4	5
X14 Improved diagnosis and treatment ability of health institutions at this level	1	2	3	4	5
X15 Regulated Management of health institutions at this level	1	2	3	4	5
X16 Increased business revenues of health institutions at this level	1	2	3	4	5
X17 Facilitate access to medical care for urban and rural residents	1	2	3	4	5
X18 Medical personnel can provide health services that meet the health needs (in quantity and quality) of the population through collaboration	1	2	3	4	5
X19 Medical staff can establish connection with more patients to have access to timely and accurate information about residents' health status.	1	2	3	4	5
X20 Reduce medical care workload of the health institution through hierarchical medical system	1	2	3	4	5
X21 Good order and system for medical care, synergistic management	1	2	3	4	5

X22 Medical staff can implement the requirement of separation of acute and chronic diseases treatment and provide patients with treatment-rehabilitation-long-term care services in continuous way.	1	2	3	4	5
X23 Medical staff has access to shared knowledge (technology, information etc.)	1	2	3	4	5
X24 Roles and responsibilities are clearly identified for medical staff in the provision of health services.	1	2	3	4	5

Supplementary Table 6 The Questionnaire of Medical Staff in Primary Health Institutions

Survey item	Likert item				
X1 Good order and system for medical care, synergistic management	1	2	3	4	5
X2 Good infrastructure (office, medical facilities)	1	2	3	4	5
X3 Good doctor-patient relationship	1	2	3	4	5
X4 Provide scientific and reasonable promotion system for medical staff	1	2	3	4	5
X5 Good organizational culture and atmosphere	1	2	3	4	5
X6 Provide necessary working conditions for medical staff	1	2	3	4	5
X7 Provide necessary living facilities for medical staff	1	2	3	4	5
X8 Preferable security, medical dispute compensation and roadside insurance provided for medical	1	2	3	4	5
X9 Facilitate access to medical care for urban and rural residents	1	2	3	4	5
X10 Reduce medical burden of patients at the grass-roots level	1	2	3	4	5
X11 Increased business revenues of health institutions at this level	1	2	3	4	5

X12 Improve diagnosis and treatment ability of health institutions at this level	1	2	3	4	5
X13 Get access to shared knowledge	1	2	3	4	5
X14 Opportunities for continuing education and further study provided by higher-level health institutions	1	2	3	4	5
X15 Medical staff from multiple institutions all can be supported and respected when providing health services synergistically	1	2	3	4	5
X16 Get access to guidance and help from experts in lead hospitals when delivering health services synergistically	1	2	3	4	5
X17 Opinions of medical staff can be valued when making decisions on important matters	1	2	3	4	5
X18 Opinions of medical staff can be valued when decisions involving the interests of medical staff	1	2	3	4	5
X19 Roles and responsibilities are clearly identified for medical staff in the provision of health services.	1	2	3	4	5
X20 Medical personnel can provide health services that meet the health needs (in quantity and quality) of the population through collaboration	1	2	3	4	5
X21 Medical staff can implement the requirement of separation of acute and chronic diseases treatment and provide patients with treatment-rehabilitation-long-term care services in continuous way.	1	2	3	4	5
X22 Increase remuneration level for medical staff	1	2	3	4	5
X23 More training provided to medical staff to improve their abilities	1	2	3	4	5
X24 Good personal development obtained by medical staff	1	2	3	4	5

Supplementary Table 7 Basic demographic characteristics of County Hospital Managers

Variable	Full sample (N=89)	Non-medical alliance group (N=31)	medical alliance group (N=58)
Gender（%）			
male	45（50.60）	20（64.52）	25（43.10）
female	44（49.40）	11（35.48）	33（56.90）
Age（year）	41.65±10.67	46.9±8.44	38.84±10.73
Politics profile（%）			
Chinese communist party members	32（36.00）	3（9.68）	29（50.00）
Non-Communist Party member	57（64.00）	28（90.32）	29（50.00）
Employment mode（%）			
permanent employees	70（78.70）	29（93.55）	41（70.69）
temporary employees	19（21.30）	2（6.45）	17（29.31）
Years of working（year）	18.78±11.07	22.13±9.42	16.98±11.53

Supplementary Table 8 Basic demographic characteristics of Managers in Primary Health Institutions

Variable	Full sample (N=448)	Non-medical alliance group (N=89)	medical alliance group (N=359)
Gender（%）			
male	281（62.72）	54（60.67）	227（63.23）
female	167（37.28）	35（39.33）	132（36.77）
Age（year）	43.57±9.61	40.93±8.89	44.22±9.67
Marital Status（%）			
Have a spouse	413（92.19）	84（94.38）	329（91.64）
mateless	35（7.81）	5（5.62）	30（8.36）
Politics profile（%）			
Chinese communist party members	152（33.93）	34（38.20）	118（32.87）
Non-Communist Party member	296（66.07）	55（61.80）	241（67.13）
Health Status（%）			
Not sick	431（96.21）	80（89.89）	351（97.77）
sicken	17（3.79）	9（10.11）	8（2.23）
work unit （%）			

Variable	Full sample	Non-medical alliance group	medical alliance group
	（N=448）	（N=89）	（N=359）
County-level medical institutions	31（6.92）	12（13.48）	19（5.29）
Township health centers / community health service centers	287（64.06）	77（86.52）	210（58.50）
Village clinic	130（29.02）	0（0.00）	130（36.21）
Employment mode（%）			
permanent employees	306（68.30）	85（95.51）	221（61.56）
temporary employees	142（31.70）	4（4.49）	138（38.44）
Years of working（year）	18.71±11.43	14.03±9.26	19.87±11.62
Years of Schooling（year）	14.57±1.26	15.10±0.99	14.43±1.28

Supplementary Table9 Basic demographic characteristics of Medical Staff in County Hospitals

Variable	Full sample	Non-medical alliance group	medical alliance group
	（N=302）	（N=80）	（N=222）
Gender（%）			
male	148（49.00）	26（32.50）	122（54.95）
female	154（51.00）	54（67.50）	100（45.05）
Age（year）	35.81±9.32	33.69±9.05	36.58±9.32
Marital Status（%）			
Have a spouse	244（80.79）	66（82.50）	178（80.18）
mateless	58（19.21）	14（17.50）	44（19.82）
Politics Profile（%）			
Chinese communist party members	90（29.80）	17（21.25）	73（32.88）
Non-Communist Party member	212（70.20）	63（78.75）	149（67.12）
Health Status（%）			
Not sick	295（97.68）	78（97.50）	217（97.75）
sicken	7（2.32）	2（2.50）	5（2.25）
Employment Mode（%）			
permanent employees	180（59.60）	54（67.50）	126（56.76）
temporary employees	122（40.40）	26（32.50）	96（43.24）
Years of working（year）	12.05±9.35	13.12±8.97	11.66±9.48
Years of Schooling（year）	15.67±0.79	15.63±0.68	15.68±0.82
Job Type（%）			

Variable	Full sample	Non-medical alliance group	medical alliance group
	（N=302）	（N=80）	（N=222）
clinician	175（57.95）	42（52.50）	133（59.91）
paramedic	92（30.46）	29（36.25）	63（28.38）
Auxiliary department personnel	10（3.31）	2（2.50）	8（3.60）
public health doctor	5（1.66）	1（1.25）	4（1.80）
other	20（6.62）	6（7.50）	14（6.31）
Vocational Qualification（%）			
Assistant practitioner	6（1.99）	0（0.00）	6（2.70）
practicing doctor	168（55.63）	42（52.50）	126（56.76）
Practicing nurse	94（31.13）	29（36.25）	65（29.28）
other	34（11.26）	9（11.25）	25（11.26）
Job Title（%）			
Yes	274（90.73）	75（93.75）	199（89.64）
No	28（9.27）	5（6.25）	23（10.36）
Average Monthly Salary（Ln value）	8.21±1.59	8.39±1.06	8.14±1.74
Salary Expectation（Ln value）	9.07±1.04	9.12±0.54	9.05±1.17
Pension（%）			
Yes	281（93.05）	77（96.25）	204（91.89）
No	21（6.95）	3（3.75）	18（8.11）

Supplementary Table10 Basic demographic characteristics of Medical Staff in Primary Health Institutions

Variable	Full sample	Non-medical alliance group	medical alliance group
	（N=1093）	（N=285）	（N=808）
Gender（%）			
male	484（44.28）	150（52.63）	334（41.34）
female	609（55.72）	135（47.37）	474（58.66）
Age（year）	38.83±10.87	37.69±10.67	39.23±10.92
Marital Status（%）			
Have a spouse	897（82.07）	239（83.86）	658（81.44）
mateless	196（17.93）	46（16.14）	150（18.56）

Variable	Full sample (N=1093)	Non-medical alliance group (N=285)	medical alliance group (N=808)
Politics Profile（%）			
Chinese communist party members	313（28.64）	97（34.04）	216（26.73）
Non-Communist Party member	780（71.36）	188（65.96）	592（73.27）
Health Status（%）			
Not sick	1044（95.52）	271（95.09）	773（95.67）
sicken	49（4.48）	14（4.91）	35（4.33）
work unit （%）			
County-level medical institutions	66（6.04）	37（12.98）	29（3.59）
Township health centers / community health service centers	970（88.75）	248（87.02）	722（89.36）
Village clinic	57（5.22）	0（0.00）	57（7.05）
Employment Mode（%）			
permanent employees	862（78.87）	242（84.91）	620（76.73）
temporary employees	231（21.13）	43（15.09）	188（23.27）
Years of working（year）	14.58±11.53	12.58±10.55	15.29±11.78
Years of Schooling（year）	14.67±1.16	14.99±1.12	14.56±1.15
Job Type（%）			
clinician	515（47.12）	195（68.42）	320（39.60）
paramedic	256（23.42）	46（16.14）	210（25.99）
Auxiliary department personnel	125（11.44）	11（3.86）	114（14.11）
public health doctor	76（6.95）	18（6.32）	58（7.18）
other	121（11.07）	15（5.26）	106（13.12）
Vocational Qualification（%）			
Assistant practitioner	217（19.85）	56（19.65）	161（19.93）
practicing doctor	369（33.76）	149（52.28）	220（27.23）
Practicing nurse	254（23.24）	41（14.39）	213（26.36）
other	253（23.15）	39（13.68）	214（26.48）
Job title（%）			
Yes	940（86.00）	256（89.82）	684（84.65）
No	153（14.00）	29（10.18）	124（15.35）
Average Monthly Salary	8.12±0.55	8.16±0.64	8.11±0.52

Variable	Full sample	Non-medical alliance group	medical alliance group
	（N=1093）	（N=285）	（N=808）
(Ln value)			
Salary Expectation (Ln value)	8.71±1.07	8.85±0.52	8.66±1.20
Pension（%）			
Yes	1006（92.04）	269（94.39）	737（91.21）
No	87（7.96）	16（5.61）	71（8.79）

Supplementary Table11 Core categories and their relational structure as formed by selective coding

**Core Code**	**Main Axis Code**	**Serial Number**	**Open Coding**	**Reference Point**
structure	Organizational Management Structure	1	Strengthening Institutional and Strategic Coordination among Healthcare Institutions at All Levels	16
		2	Reasonable Mechanisms for Shared Interests	22
		3	Corresponding Assessment Mechanisms and Incentive Systems	15
	Division of Labor Collaboration Mechanism	4	Reasonable Mobility Mechanism for Professional and Technical Talents at All Levels of Healthcare Institutions	20
		5	Clearly Defined Responsibilities, Rights, and Power Relationships among Healthcare Service Institutions at All Levels	13
	Synergistic Development Philosophy	6	A Positive Organizational Culture	28
		7	Sound Diagnostic and Collaborative Management Orders and Systems	26
process	Integration of Human, Financial, and Material Resources	8	Integrated Management of Human, Financial, and Material Resources, Effectively Promoting the Vertical Integration of the Healthcare Service System, and Facilitating Resource Decentralization	13
		9	Good Personal Development Opportunities	18
		10	Scientific and Reasonable Promotion System	22

		11	Human, Financial, and Material Resources Adjusted Timely According to the Development of Healthcare Institutions	10
		12	Smooth Communication Channels within the Healthcare Service System	13
	Information Sharing Mechanism	13	Access to Shared Knowledge (Technology, Information, etc.)	12
		14	Healthcare personnel receive more training to enhance their capabilities	8
		15	Mutual recognition of examination results within the healthcare service system	14
	Seamless Vertical Integration of Medical Resources	16	High-quality information system for the healthcare service system	12
		17	County-level healthcare talents are decentralized to grassroots healthcare service institutions	8
		18	Enhancing the Accessibility of Medical Care for Residents	13
	Continuity of Healthcare Services	19	Strengthening the Continuity of Services between Healthcare Institutions through Bidirectional Referral and Medication, Improving the Consultation Rate within the County	5
Outcome		20	Providing Patients with Continuous Services for Disease Diagnosis, Treatment, Rehabilitation, and Long-term Care	8
		21	Enhancing the Service Capabilities of Healthcare Personnel in Primary Healthcare Institutions	8
	Medical Service Capability	22	Improving the Diagnosis and Treatment Capabilities of Primary Healthcare Institutions	5
		23	County-level Healthcare Service Institutions playing a driving role, effectively enhancing the capabilities for diagnosing and treating critical and severe cases	5

Supplementary Table 12 Results of Factor Analysis of Lead County Hospital Managers' Satisfaction with Cross-institutional Synergistic Development

Item	Rotated factor loading matrix		Factor score coefficient matrix	
	F_1	F_2	F_1	F_2

Item	Rotated factor loading matrix		Factor score coefficient matrix	
	F_1	F_2	F_1	F_2
X1 Establish a referral system for healthcare services	0.69	/	0.13	0.08
X2 Mutual transmission, circulation and recognition of business information	0.78	/	0.31	-0.13
X3 Guarantee continuity of two-way referral medications	0.77	/	0.35	-0.19
X4 Bring into play the radiation role of lead hospitals//county-level health institutions	0.84	/	0.46	-0.32
X5 Improve emergency and critical care capability of lead hospitals/county-level health institutions	0.82	/	0.43	-0.28
X6 Establish unified governance organization or coordination department	/	0.81	-0.43	0.72
X7 Adjust and train human resources in timely manner along with health institution development	/	0.80	-0.32	0.61
X8 Adjust and allocate health facilities and technologies among health institutions in unified manner based on resources allocation plan and business development plan	/	0.67	-0.12	0.36

Note: a. The explained variances of the 2 factors were 45.044% and 34.068%, respectively, with a cumulative explained variance of 79.112%.

b. The 2 factors were assigned according to their corresponding loading values of the questions in this factor to form 2 new variables Yi (i=1, 2), Y1=0.69X1+0.78X2+0.77X3+0.84X4+0.82X5/ (0.69+0.78+0.77+0.84+0.82), and Y2 is obtained similarly.

Based on the factor score coefficient matrix, the factor score function was constructed:

F1=0.13X1+0.31X2+0.35X3+0.46X4+0.43X5-0.43X6-0.32X7-0.12X8;

F2=0.08X1-0.13X2-0.19X3-0.32X4-0.28X5+0.72X6+0.61X7 +0.36X8.

c. Overall satisfaction score:

F=(45.04%F1+34.07%F2)/79.11%=0.11X1+0.12X2+0.12X3+0.13X4+0.13X5+0.06X6+0.08X7+ 0.09X8. To derive the actual satisfaction of the lead hospital managers, the coefficients of the

variables in the factor score model were used to determine the original actual weights of the variables in the model, and then the original values of the variables were substituted to obtain the satisfaction values. Actual weights of variables =Ci/$\sum_{i=1}^{8} ci$. The actual satisfaction score: F=0.13X1+0.15X2+0.14X3+0.15X4+0.15X5+0.08X6+0.09X7+0.11X8.

Supplementary Table 13 Results of Factor Analysis of Satisfaction with Cross-Institutional Synergy among Managers of Primary Health Institutions

Item	Rotated factor loading matrix		Factor score coefficient matrix	
	F_1	F_2	F_1	F_2
X1 Bring into play the radiation role of lead hospitals/county-level health institutions	0.85	/	0.33	-0.26
X2 Improve emergency and critical care capability of health institutions at this level	0.81	/	0.29	-0.22
X3 Awareness of holism and collaboration	0.81	/	0.21	-0.12
X4 Integrated-service concept and group consciousness	0.80	/	0.23	-0.14
X5 Establish and improve Benefit-sharing mechanism	0.79	/	0.20	-0.10
X6 Guarantee continuity of two-way referral medications	0.76	/	0.20	-0.11
X7 Improve organizational performance of member institutions/primary health institutions	0.73	/	0.14	-0.03
X8 Mutual transmission, circulation and recognition of business information	0.69	/	0.11	0.01
X9 Establish healthcare referral system	0.69	/	0.07	0.05
X10 Adjust capital investment in timely manner in line with development needs of health institutions	/	0.87	-0.34	0.55
X11 Adjust government financial inputs in timely manner in line with the development needs of health	/	0.87	-0.35	0.56

Item	Rotated factor loading matrix		Factor score coefficient matrix	
	F_1	F_2	F_1	F_2
institutions at all levels				
X12 Adjust and allocate health facilities and technologies among health institutions in unified manner based on resources allocation plan and business development plan	/	0.74	-0.16	0.34
X13 Adjust and train human resources in timely manner along with health institution development	/	0.67	-0.07	0.22
X14 Establish unified governance organization or coordination department, improve organizational structure and collaboration mechanism, and form unified governance structure	/	0.85	-0.04	0.36

Note: The explained variances of the 2 factors were 47.510% and 34.290%, respectively, with a cumulative explained variance of 83.800%.

Supplementary Table 14 Results of Factor Analysis of Satisfaction with Cross-Institutional Synergy Satisfaction among the Medical Staff in County Hospitals

Item	Rotated Factor Loading Matrix		Factor Score Coefficient Matrix	
	F_1	F_2	F_1	F_2
X1 Provide necessary living facilities for medical staff	0.85	/	0.28	-0.22
X2 Good personal development obtained by medical staff	0.82	/	0.22	-0.15
X3 More training provided to medical staff to improve their abilities	0.82	/	0.24	-0.18
X4 Preferable security, medical dispute compensation and roadside insurance provided for medical staff	0.77	/	0.18	-0.12
X5 Opinions of medical staff can be valued when decisions	0.77	/	0.15	-0.08

Item	Rotated Factor Loading Matrix		Factor Score Coefficient Matrix	
	F_1	F_2	F_1	F_2
involving the interests of medical staff				
X6 Increase the remuneration level for medical staff	0.75	/	0.21	-0.15
X7 Medical staff from multiple institutions all can be supported and respected when providing health services synergistically	0.74	/	0.13	-0.06
X8 Provide necessary working conditions for medical staff	0.74	/	0.14	-0.07
X9 Opinions of medical staff can be valued when making decisions on important matters	0.73	/	0.11	-0.04
X10 Good organizational culture and atmosphere	0.72	/	0.13	-0.06
X11 Good doctor-patient relationship	0.64	/	0.09	-0.03
X12 Scientific and reasonable promotion system provided for medical staff	0.61	/	0.04	0.03
X13 Reduce medical burden of patients at grass-roots level	/	0.85	-0.20	0.27
X14 Improved diagnosis and treatment ability of health institutions at this level	/	0.84	-0.19	0.26
X15 Regulated Management of health institutions at this level	/	0.82	-0.16	0.23
X16 Increased business revenues of health institutions at this level	/	0.81	-0.19	0.26
X17 Facilitate access to medical care for urban and rural residents	/	0.79	-0.14	0.21
X18 Medical personnel can provide health services that meet the health needs (in quantity and quality) of the population through collaboration	/	0.73	-0.05	0.13
X19 Medical staff can establish connection with more patients to have access to timely and accurate information about	/	0.73	-0.07	0.14

Item	Rotated Factor Loading Matrix		Factor Score Coefficient Matrix	
	F_1	F_2	F_1	F_2
residents' health status.				
X20 Reduce medical care workload of the health institution through hierarchical medical system	/	0.73	-0.07	0.14
X21 Good order and system for medical care, synergistic management	/	0.68	-0.03	0.10
X22 Medical staff can implement the requirement of separation of acute and chronic diseases treatment and provide patients with treatment-rehabilitation-long-term care services in continuous way.	/	0.68	-0.01	0.08
X23 Medical staff has access to shared knowledge (technology, information etc.)	/	0.66	0.02	0.05
X24 Roles and responsibilities are clearly identified for medical staff in the provision of health services.	/	0.66	0.03	0.04

Note: The explained variances of the 2 factors were 41.706% and 40.159%, respectively, with a cumulative explained variance of 81.865%.

Supplementary Table15 Results of Factor Analysis of Satisfaction with Cross-Institutional Synergy among Medical Staff of Primary Health Institutions

Item	Rotated Factor Loading Matrix				Factor Score coefficient matrix			
	F_1	F_2	F_3	F_2	F_1	F_2	F_3	F_4
X1 Good order and system for medical care, synergistic management	0.78	/	/	/	0.44	-0.15	0.00	-0.26
X2 Good infrastructure (office, medical facilities)	0.75			/	0.38	-0.12	-0.18	-0.02
X3 Good doctor-patient relationship	0.71			/	0.35	-0.02	-0.09	-0.20
X4 Provide scientific and reasonable promotion system for	0.69			/	0.35	-0.24	0.06	-0.13

Item	Rotated Factor Loading Matrix				Factor Score coefficient matrix			
	F_1	F_2	F_3	F_2	F_1	F_2	F_3	F_4
medical staff								
X5 Good organizational culture and atmosphere	0.69			/	0.29	-0.07	-0.03	-0.13
X6 Provide necessary working conditions for medical staff	0.59			/	0.13	0.05	-0.29	0.26
X7 Provide necessary living facilities for medical staff	0.58	/	/	/	0.13	0.00	-0.30	0.32
X8 Preferable security, medical dispute compensation and roadside insurance provided for medical	0.53	/	/	/	0.08	0.07	-0.23	0.21
X9 Facilitate access to medical care for urban and rural residents	/	0.74		/	-0.05	0.48	-0.24	-0.13
X10 Reduce medical burden of patients at the grass-roots level	/	0.73	/	/	-0.12	0.47	-0.29	0.04
X11 Increased business revenues of health institutions at this level	/	0.70	/	/	-0.20	0.43	-0.25	0.15
X12 Improve diagnosis and treatment ability of health institutions at this level	/	0.67	/	/	-0.08	0.31	-0.04	-0.12
X13 Get access to shared knowledge	/	0.60	/	/	-0.06	0.20	0.06	-0.12
X14 Opportunities for continuing education and further study provided by higher-level health institutions	/	/	0.69	/	-0.11	-0.29	0.42	0.09
X15 Medical staff from multiple institutions all can be supported and respected when providing health services synergistically	/	/	0.68	/	-0.04	-0.15	0.38	-0.12
X16 Get access to guidance and help from experts in lead hospitals when delivering health services synergistically	/		0.68	/	-0.07	-0.17	0.39	-0.06
X17 Opinions of medical staff can be valued when making decisions on important matters	/	/	0.61	/	-0.08	-0.02	0.24	-0.05
X18 Opinions of medical staff can be valued when decisions involving the interests of medical	/	/	0.61	/	-0.08	-0.01	0.24	-0.07

Item	Rotated Factor Loading Matrix				Factor Score coefficient matrix			
	F_1	F_2	F_3	F_2	F_1	F_2	F_3	F_4
staff								
X19 Roles and responsibilities are clearly identified for medical staff in the provision of health services.	/	/	0.60	/	-0.04	0.03	0.24	-0.18
X20 Medical personnel can provide health services that meet the health needs (in quantity and quality) of the population through collaboration	/	/	0.58	/	-0.06	0.10	0.19	-0.16
X21 Medical staff can implement the requirement of separation of acute and chronic diseases treatment and provide patients with treatment-rehabilitation-long-term care services in continuous way.	/	/	0.57	/	-0.06	0.13	0.18	-0.19
X22 Increase remuneration level for medical staff	/	/	/	0.77	-0.22	-0.09	0.17	0.72
X23 More training provided to medical staff to improve their abilities	/	/	/	0.60	-0.12	-0.21	0.13	0.37
X24 Good personal development obtained by medical staff	/	/	/	0.58	-0.08	-0.15	0.07	0.33

Note: The explained variances of the four factors were 24.005%, 22.517%, 22.376%, and 15.119%, respectively, with a cumulative explained variance of 84.016%.

Supplementary Table16 Propensity Score Matching Quality Test for Satisfaction with Cross-institutional Synergy among Managers of Lead County Hospital Managers

Variable	matching status	average value		Standard Error （%）	Error Reduction （%）	T-Test		Logit
		Control Group	Treatment Group			T Value	P>|t|	
Gender	Before matching	0.645	0.431	-43.40	53.30	-1.94	0.055	-1.187*
	After matching	0.376	0.476	20.30		-0.64	0.524	（0.717）
Age	Before matching	46.903	38.845	-83.50	87.10	-3.62	<0.001	-0.433***
	After matching	41.580	42.619	10.80		-0.40	0.688	（0.146）

Variable	matching status	average value: Control Group	average value: Treatment Group	Standard Error （%）	Error Reduction （%）	T-Test: T Value	T-Test: P>|t|	Logit
Politics profile	Before matching	0.097	0.500	97.10	65.70	4.07	<0.001	3.048***
	After matching	0.100	0.238	33.30		1.19	0.243	（0.813）
Employment mode	Before matching	0.936	0.707	-61.80	96.00	-2.57	0.012	-0.175
	After matching	0.800	0.810	2.50		0.07	0.942	（1.139）
working years	Before matching	22.129	16.983	-48.90	96.80	-2.13	0.036	0.311**
	After matching	19.404	19.238	-1.60		-0.06	0.956	（0.120）

Supplementary Table 17 Propensity Score Matching Quality Test for Satisfaction with Cross-institutional Synergy among Managers of Primary Health Institutions

Variable	matching status	average value: Control Group	average value: Treatment Group	Standard Error （%）	Error Reduction （%）	T-Test: T Value	T-Test: P>|t|	Logit
Gender	Before matching	0.607	0.632	5.20	33.40	0.45	0.656	-0.495
	After matching	0.612	0.537	-3.50		-0.36	0.721	（0.308）
Age	Before matching	40.933	44.223	35.40	86.20	2.91	0.004	0.008
	After matching	40.822	41.275	4.90		0.53	0.598	（0.025）
Marital Status	Before matching	0.944	0.916	-10.70	84.00	-0.86	0.390	-1.009*
	After matching	0.885	0.904	7.40		0.64	0.525	（0.562）
Politics profile	Before matching	0.382	0.328	-11.10	40.20	-0.95	0.343	-0.011
	After matching	0.336	0.367	6.60		0.70	0.487	（0.297）
Health Status	Before matching	0.899	0.978	33.10	96.00	3.52	<0.001	1.928***
	After matching	0.976	0.973	-1.30		-0.21	0.834	（0.614）

Variable	matching status	average value		Standard Error（%）	Error Reduction（%）	T-Test		Logit
		Control Group	Treatment Group			T Value	P>|t|	
Employer	Before matching	1.865	2.309	94.80	93.60	7.08	<0.001	1.306***
	After matching	1.903	1.931	6.10		0.97	0.335	（0.371）
Employment mode	Before matching	0.955	0.616	-90.60	91.20	-6.43	<0.001	-1.440**
	After matching	0.924	0.951	8.00		1.31	0.191	（0.576）
working years	Before matching	14.034	19.872	55.60	58.20	4.40	<0.001	0.044**
	After matching	15.783	16.243	4.40		0.46	0.647	（0.020）
Years of Schooling	Before matching	15.101	14.935	-58.20	79.40	-4.58	0.000	-0.089
	After matching	15.128	14.991	-12.00		-1.39	0.165	（0.152）

Note: 1. The corresponding standard errors are in parentheses, ***P<0.01, **P<0.05, respe -ctively.

2. The sample matching results show that, after matching, the absolute values of standard deviations of variables are less than 15%, and the matching effect is good.

Supplementary Table 18 Propensity Score Matching Quality Test for Satisfaction with Cross-institutional Synergy among County Hospital Medical Staff

Viable	Matching Status	Average Value		Standard Error（%）	Error Reduction（%）	t Testing		*Logit*
		Control Group	Treatment Group			t Value	*P*>|*t*|	
Gender	Before matching	0.325	0.550	46.30	92.80	3.50	0.001	0.810**
	After matching	0.555	0.539	-3.30		-0.34	0.736	（0.298）
Age	Before matching	33.688	36.577	31.50	79.30	2.40	0.017	0.034*
	After matching	36.824	36.226	-6.50		-0.63	0.531	（0.021）
Marital Status	Before matching	0.825	0.802	-5.90	32.70	-0.45	0.653	-0.803*
	After matching	0.791	0.806	4.00		0.40	0.686	（0.437）
Politics Profile	Before matching	0.213	0.329	26.30	93.70	1.96	0.051	0.464
	After	0.321	0.314	-1.70		-0.16	0.869	（0.341）

Viable	Matching Status	Average Value		Standard Error （%）	Error Reduction （%）	t Testing		*Logit*
		Control Group	Treatment Group			t Value	*P*>|*t*|	
	matching							
Health Status	Before matching	0.975	0.977	1.60	-168.50	0.13	0.900	1.069
	After matching	0.984	0.977	-4.30		-0.50	0.619	（0.965）
Employment Mode	Before matching	0.675	0.568	-22.20	89.90	-1.68	0.094	-0.592*
	After matching	0.583	0.571	-2.20		-0.23	0.819	（0.338）
Working years	Before matching	13.125	11.658	-15.90	69.80	-1.20	0.230	0.002
	After matching	11.381	11.825	4.80		0.52	0.606	（0.018）
Years of Schooling	Before matching	15.625	15.685	7.90	-58.50	0.58	0.562	0.215
	After matching	15.601	15.696	12.50		1.26	0.209	（0.194）
Job Type	Before matching	1.750	1.662	-8.00	80.30	-0.62	0.536	-0.023
	After matching	1.623	1.641	1.60		0.17	0.864	（0.189）
Vocational Qualification	Before matching	2.588	2.491	-13.60	47.80	-1.03	0.304	-0.358
	After matching	2.525	2.475	-7.10		-0.74	0.458	（0.280）
Job Title	Before matching	0.938	0.896	-14.90	60.30	-1.09	0.279	-0.584
	After matching	0.920	0.903	-5.90		-0.60	0.551	（0.661）
Average Monthly Salary	Before matching	8.390	8.142	-17.20	33.90	-1.20	0.232	-0.020
	After matching	8.340	8.177	-11.40		-1.24	0.215	（0.178）
Salary Expectation	Before matching	9.121	9.047	-8.00	58.20	-0.54	0.591	0.046
	After matching	9.090	9.059	-3.40		-0.34	0.734	（0.218）
Pension	Before matching	0.963	0.919	-18.50	73.80	-1.31	0.190	-0.635
	After matching	0.947	0.935	-4.80		-0.50	0.614	（0.776）

Note: 1. The corresponding standard errors are in parentheses; **P<0.05, *P<0.1, respectively.

2. The sample matching results show that the standard deviation is less than 10% in absolute value after matching all variables except years of schooling and average monthly salary, and the matching effect is good.

Supplementary Table 19 Propensity score Matching Quality Test for Satisfaction with Cross-institutional Synergy among Medical Staff of Primary Health Institutions

Viable	Matching Status	Average Value		Standard Deviation （%）	Error Reduction （%）	t Test		*Logit*
		Control Group	Treatment Group			t Value	*P*>|*t*|	
Gender	Before matching	0.526	0.413	-22.80	79.10	-3.31	0.001	-0.371**
	After matching	0.378	0.402	4.70		0.94	0.345	（0.169）
Age	Before matching	37.695	39.225	14.20	45.60	2.05	0.041	-0.013
	After matching	37.532	38.365	7.70		1.53	0.126	（0.014）
Marital Status	Before matching	0.839	0.814	-6.40	40.60	-0.92	0.360	-0.366
	After matching	0.792	0.806	3.80		0.70	0.483	（0.227）
Politics Profile	Before matching	0.340	0.267	-15.90	87.50	-2.35	0.019	-0.390**
	After matching	0.275	0.266	-2.00		-0.40	0.687	（0.166）
Health Status	Before matching	0.951	0.957	2.80	77.50	0.41	0.684	0.207
	After matching	0.959	0.958	-0.60		-0.13	0.898	（0.365）
Employer	Before matching	1.870	2.035	49.70	86.50	7.28	<0.001	1.524***
	After matching	1.969	1.991	6.70		1.97	0.049	（0.271）
Employment Mode	Before matching	0.849	0.767	-20.90	58.40	-2.92	0.004	-0.176
	After matching	0.762	0.796	8.70		1.60	0.109	（0.233）
Years of Service	Before matching	12.582	15.290	24.20	44.90	3.43	0.001	0.034***
	After matching	12.741	14.233	13.30		2.60	0.010	（0.013）
Years of Schooling	Before matching	14.993	14.556	-38.50	79.20	-5.56	<0.001	-0.108

Viable	Matching Status	Average Value		Standard Deviation (%)	Error Reduction (%)	t Test		*Logit*
		Control Group	Treatment Group			t Value	*P*>|*t*|	
	After matching	14.727	14.636	-8.00		-1.56	0.119	（0.081）
Job Type	Before matching	1.639	2.282	50.50	76.50	7.02	<0.001	0.305***
	After matching	2.319	2.168	-11.90		-2.09	0.037	（0.078）
Vocational Qualification	Before matching	2.221	2.594	37.20	71.80	5.20	<0.001	0.123
	After matching	2.647	2.542	-10.50		-1.95	0.052	（0.092）
Job title	Before matching	0.898	0.847	-15.50	74.70	-2.17	0.031	0.125
	After matching	0.848	0.861	3.90		0.73	0.468	（0.258）
Average Monthly Salary	Before matching	8.160	8.112	-8.20	-9.70	-1.25	0.211	0.267*
	After matching	8.176	8.124	-9.00		-2.23	0.026	（0.150）
Salary Expectation	Before matching	8.853	8.658	-21.10	74.70	-2.66	0.008	-0.161
	After matching	8.793	8.744	-5.30		-1.46	0.144	（-0.112）
Pension	Before matching	0.944	0.912	-12.30	87.60	-1.70	0.089	0.317
	After matching	0.942	0.938	-1.50		-0.32	0.746	（0.336）

Note: 1. The corresponding standard errors are in parentheses; ***P<0.01, and *P<0.1, **P<0.05respectively.

2. The sample matching results show that the standard deviation is less than 10% in absolute value after matching all variables except years of schooling and average monthly salary, and the matching effect is good.
